# Antimicrobial stewardship programs in primary and secondary care settings in India: current challenges, facilitators, perceptions, and impact – a scoping review

**DOI:** 10.1186/s12879-025-11851-0

**Published:** 2025-11-11

**Authors:** Rajalakshmi Rajendran, Shravya Chitrapady, MU Tejashree, Kapu Haritha, Jayaraj Mymbilly Balakrishnan, Sreedharan Nair, Mohammed Salim Karattuthodi, Sohil Khan, Sneh Shalini, Rangaswamy Uday Kumar, Nettoor Veettil Vajid, Girish Thunga

**Affiliations:** 1 Department of Pharmacy Practice, Manipal College of Pharmaceutical Sciences, Manipal Academy of Higher Education, Manipal, 576104 Karnataka India; 2 Department of Emergency Medicine, Kasturba Medical College and Hospital, Manipal Academy of Higher Education, Manipal, 576104 Karnataka India; 3 Indian Council of Medical Research, New Delhi, India; 4 Department of Pharmacy Practice, Amrita School of Pharmacy, Amrita Vishwa Vidyapeetham, Kochi, 682041 Kerala India; 5 Department of Research and Development, IQRAA International hospital and Research Centre, Calicut, Kerala India; 6 Pharmacotherapeutics and Evidence Based Practice, School of Pharmacy and Medical Sciences, Griffith University, Gold Coast, Queensland,, Australia; 7 Centre for Toxicovigilance and Drug Safety, Manipal College of Pharmaceutical Sciences, Manipal Academy of Higher Education, Manipal, 576104 Karnataka India

**Keywords:** Antimicrobial resistance, Antimicrobial stewardship, Evidence synthesis, Primary care, Secondary care hospital, Facilitators, Barriers

## Abstract

**Introduction:**

Antimicrobial stewardship programs (AMSP) is a major initiative of World Health Organization Global Action Plan (WHO-GAP) to address antimicrobial resistance (AMR). The program aims to promote the rational use of antibiotics to help improve patient outcomes. Existing evidence on AMSPs and their activities is largely restricted to the resource intense settings in India thereby impacting sustainability and adaptation of initiatives within rural/remote region.

**Aims:**

The review focus on the current state of AMSP practices in the Indian primary and secondary care settings, identifying the challenges, enablers, perceptions and practice of physicians, and the impact of current AMSP interventions.

**Methods:**

We conducted electronic database searches on PubMed, Scopus, and EMBASE, in adherence to the PRISMA-ScR guidelines. Our review identifies four key domains such as barriers to AMSP establishment, facilitators for establishing AMSP, AMSP perceptions and practices among the prescribers, and the impact of AMSP interventions.

**Results:**

Among the 4739 articles searched, five studies met the inclusion criteria, in the primary and secondary care clinical settings in India. We focused on articles published after the 2015 post WHO GAP implementation. Barriers to AMSP implementation, including access to the healthcare system, poor regulatory implementation, poor knowledge and awareness about antibiotic regulations and AMR, and lack of awareness in all the studies. Facilitators identified for hospital AMSP implementation include, use of staff engagement, audit feedback and discussion, administrative representation, documentation, interactive sessions and education. Only one study could explore the impacts of AMSP intervention on both clinical and microbiological outcomes. The major outcomes included more appropriate antibiotic prescriptions, reduced consumption of antimicrobials, reduced hospital stays and decrease in mortality rate.

**Conclusion:**

The existing evidence based on adaptation and implementation of AMSP is sparse. This study highlights the urgent need for widespread AMSP in the resource-constraint settings of India. Multipronged approach is crucial to encompass skilled human resources, implement facility-specific interventions, develop accessible guidelines for antibiotic use, providing economic support and increasing awareness through capacity-building programs, and can attain effective establishment of AMSPs.

**Supplementary Information:**

The online version contains supplementary material available at 10.1186/s12879-025-11851-0.

## Introduction

Recent studies from the World Health Organization (WHO) rank antimicrobial resistance (AMR) among the top ten public health threats [1, 2]. According to 2019 statistics, AMR directly caused 1.3 million deaths [3, 4]. If this situation persists, experts estimate that the mortality rate may increase to 10 million by 2050, with 90% of fatalities occurring in developing countries like India [4]. Apart from the negative impact on health, AMR also entails substantial financial costs for patients and society at large. The World Bank’s projections indicate that AMR might lower gross domestic product (GDP) by 1.1–3.8% over the course of the next 25 years, necessitating the need for US$9 billion to address the issue [5, 6].

The primary drivers of AMR in developing countries include overprescribing and inappropriate use of antibiotics [7], poor implementation of regulations [8], poor knowledge and awareness about antibiotic regulations and AMR, self-medication, patient demand, poor infection control practices and lack of antimicrobial stewardship program (AMSP) [9]. In India, AMR has emerged as a critical public health challenge, driven by a combination of widespread antibiotic misuse, easy over-the-counter access to antimicrobials, and limited enforcement of prescription regulations [10]. Studies have shown alarmingly high levels of resistance to commonly used antibiotics, including third-generation cephalosporins, fluoroquinolones, and carbapenems, particularly among pathogens such as *Escherichia coli*, *Klebsiella pneumoniae*, and *Acinetobacter baumannii* [10]. The Indian Network for Surveillance of Antimicrobial Resistance (INSAR) and reports from the Indian Council of Medical Research (ICMR) have consistently documented increasing resistance trends in both community-acquired and hospital-acquired infections [11]. In rural and semi-urban areas, the situation is exacerbated by a lack of diagnostic facilities, inadequate infection control measures, and the absence of structured AMSP [10, 11]. In order to tackle AMR, the WHO launched the Global Action Plan (GAP) in 2015 [12], and one of the main strategies to limit the improper use of antibiotics is AMSP [13, 14]. AMSP involves key set of evidence-based multidisciplinary activities to promote the rational use of antibiotics [14, 15]. AMSP is considered as a fundamental strategy that comprises of both antibiotic prescribing practices and infection control measures to combat AMR in the hospital settings [16], by promoting rational use of antibiotics improving patient outcome and ultimately contributing to considerable reduction in the healthcare cost [17, 18]. Similarly, India has undertaken several important initiatives in consistent with WHO-GAP. The ICMR has implemented AMSP models in tertiary care hospitals, developed standard treatment guidelines, and established a national AMR surveillance network. The National Action Plan on AMR (NAP-AMR), launched in 2017, outlines a multi-sectoral strategy to contain AMR [11]. Several private healthcare networks and state governments have adopted stewardship programs focusing on prescriber education, audit-and-feedback mechanisms, and restrictive antibiotic policies. However, these efforts are having limited penetration into primary and secondary healthcare facilities, where the need is often greatest.

AMSP interventions are multifactorial and complex and vary depending upon the infrastructure of the clinical settings [19, 20]. The range of interventions vary from persuasive (education and training), enabling (providing or developing facility-specific guidelines), structural (introduction of point-of-care tests or diagnostic facilities) to restrictive (formulary restriction and pre-authorisation) [2, 18, 21]. The key requirements for the effective implementation of AMSP include a dedicated and committed leadership, organisational and governance support, drug expertise, prescriber training and accountability [15]. Despite global and national progress, resource-limited settings in India continue to lag in AMSP adoption. Scaling up AMSP implementation in these settings remains a critical next step for tackling AMR in India effectively. Most recent evidence on AMSP originates from tertiary care settings [22, 23], which are typically better resourced in terms of infrastructure, personnel, and diagnostic support. In contrast, primary and secondary care facilities, which often operate under significant resource constraints, have limited representation in the existing literature [24, 25].

This scoping review aims to explore the challenges, facilitators, and outcomes of AMSP implemented in general practice settings in India. The general practice settings in India are considered for this review that include primary care, defined as the first point of contact in the healthcare system, including outpatient clinics and general practitioners operating at the community level and secondary care, defined as facilities offering specialized services through district hospitals or equivalent institutions, typically including inpatient services but lacking the highly specialized infrastructure of tertiary care hospitals. Tertiary care, which includes advanced diagnostic and treatment capabilities often available in teaching hospitals and large urban centers, was not considered in order to maintain focus on more resource-constrained settings. The review mainly targets on four domains: (1) challenges in implementing AMSP, (2) facilitators identified in the established centres, (3) the perception and behaviour of prescribers on AMSP and the (4) impact of AMS interventions in the resource-limited clinical settings in India. Previous research focused on the various AMS interventions deployed in the clinical settings, especially in the low- and middle-income countries (LMICs) [12, 17, 26]. This scoping review will help key AMS stakeholders and policymakers to understand and establish methodological strategies to overcome the challenges and identify the key areas of focus for the effective implementation of AMSP in the Indian clinical setting. It also helps the key stakeholders to understand the prescriber attitude and behaviour, the perception influencing the implementation of AMS in the Indian general practice settings.

### Research question

This scoping review aims to explore the current landscape of AMSP activities initiated in general practice settings in India.

The main research question is “What are the challenges, facilitators, prescriber perceptions, and impacts associated with the implementation of AMSPs in general practice settings in India?”

The study adopted a PIO (Population, Intervention and Outcome) format to align the research question with the study selection criteria.

*Population (P):* General practice settings and healthcare providers in India.

*Intervention (I):* Implementation of AMSPs*.*

*Outcome (O):* Identified challenges and facilitators in AMSP implementation, prescriber perceptions and behaviours, and the impact of AMS interventions.

## Methods

The study methodology adapted the framework guided by the Arksey and O’Malley [27], modified by Levac et al. [28]. The Preferred Reporting Items for Systematic review and Meta-analysis (PRISMA-ScR) criteria are followed in this scoping review [29]. To help with the process, a scoping review protocol was developed and can be obtained from the corresponding author on request.

### Search strategy

We developed a comprehensive search approach using all of the potential medical subject headings (MeSH) and keywords from previously published studies, including “antimicrobial stewardship,” “antimicrobial resistance”, “community healthcare,” “general practice,” and “public health center”. We retrieved articles from databases such as PubMed, SCOPUS and Embase. We focused on articles published after 2015, the year the WHO-GAP was initiated [13]. This led to an impact where hospital based AMSP interventions and publications gained more importance [10, 24]. We considered only articles published in English language for inclusion. Additionally, only research conducted in Indian settings were included identifying the scope of the study. In order to prevent missing studies because of various terms assigning the same word in separate databases, we employed the truncation search approach [*]. To avoid missing any more information, we also performed Google searches on the included studies’ bibliographies, pertinent works published in the same field, and relevant terms. This was done to ensure that no potentially relevant studies were missed and to capture grey literature or materials that may not be indexed in standard databases. The *Supplementary file* contains a thorough search technique across several databases.

### Study inclusion criteria

A well-defined inclusion and exclusion criteria were established to select the relevant studies, based on the domains identified.

#### Study types

To evaluate the implementation of the AMS program in general practice settings in India, quantitative research including randomized controlled trials (RCTs), cluster RCTs (cRCTs), quasi-experimental studies, cross-sectional studies, and interventional studies were included. In addition, the qualitative studies that assessed the barriers, facilitators and prescriber perception on implementing antimicrobial stewardship in community care settings were also included. We excluded studies that were conducted in tertiary care centers, overseas, non-English literature, case studies, narrative reviews, editorials, conference papers, and special reports on AMSP.

#### Intervention type and outcome

The included studies involve those asses the barriers, challenges, facilitators and perception of prescribers on the antimicrobial stewardship implementation in the primary and secondary care settings of India. It also included studies that evaluate the effectiveness of AMS activities implemented in clinical settings. Studies that assess the antimicrobial usage, prevalence of multidrug resistance infection, clinical, economical and microbiological outcome were also included, if any. Only studies related to human health were included; those focusing solely on antifungal or antiviral stewardship, or antimicrobial stewardship interventions in animal husbandry or agriculture, were excluded.

### Study selection and data extraction

Using the designated keywords to obtain publications, three authors independently assessed each article’s title and abstract before doing a full-text search to confirm the study’s eligibility. Using Microsoft Excel, three review authors independently extracted the data from the included studies using a data extraction sheet. The extracted data included articles refined to the pre-defined domains such as barriers to the implementation of AMSP, facilitators that aid in establishing the AMSP, perceptions and practices of prescribers or healthcare professionals towards AMSP and the impact of established AMSP interventions. The retrieved data included the name of the first author, year of study, place of study, type of study design, study population and the outcome measures of the study (Table 1). Barriers or challenges faced in implementing AMSP and the facilitators aiding for establishing AMSP were again subdivided based on the key influencing factors identified from the articles respectively (Table 2). The perception and practices of healthcare professionals towards AMSP were divided into knowledge, attitude and practice (KAP) (Table 3). The impact of AMSP interventions is further classified into antibiotic prescribing practice, antibiotic consumption (days of therapy (DOT) or defined daily dose (DDD)), clinical outcome (mortality due to hospital acquired infection (HAI), prolonged hospital stays), microbiological outcome (prevalence of multidrug resistant organism (MDRO), surgical site infection rates) and economic outcomes (cost of treatment, antibiotic consumption costs) (Table 4). Antibiotic consumption refers to the amount of antibiotics consumed in accordance with the pharmacy consumption record, while antibiotic prescribing refers to the quality of antibiotic use (appropriateness of the prescription, duration of treatment, use of broad spectrum and costlier antibiotics when narrow spectrum, cheaper alternatives are available) [30]. We also tried to incorporate the limitations of the studies that described the impact of AMSP interventions. Disagreements that developed during the selection of the study and the extraction of the data were settled by agreement or conversation with another researcher.

**Table 1:**
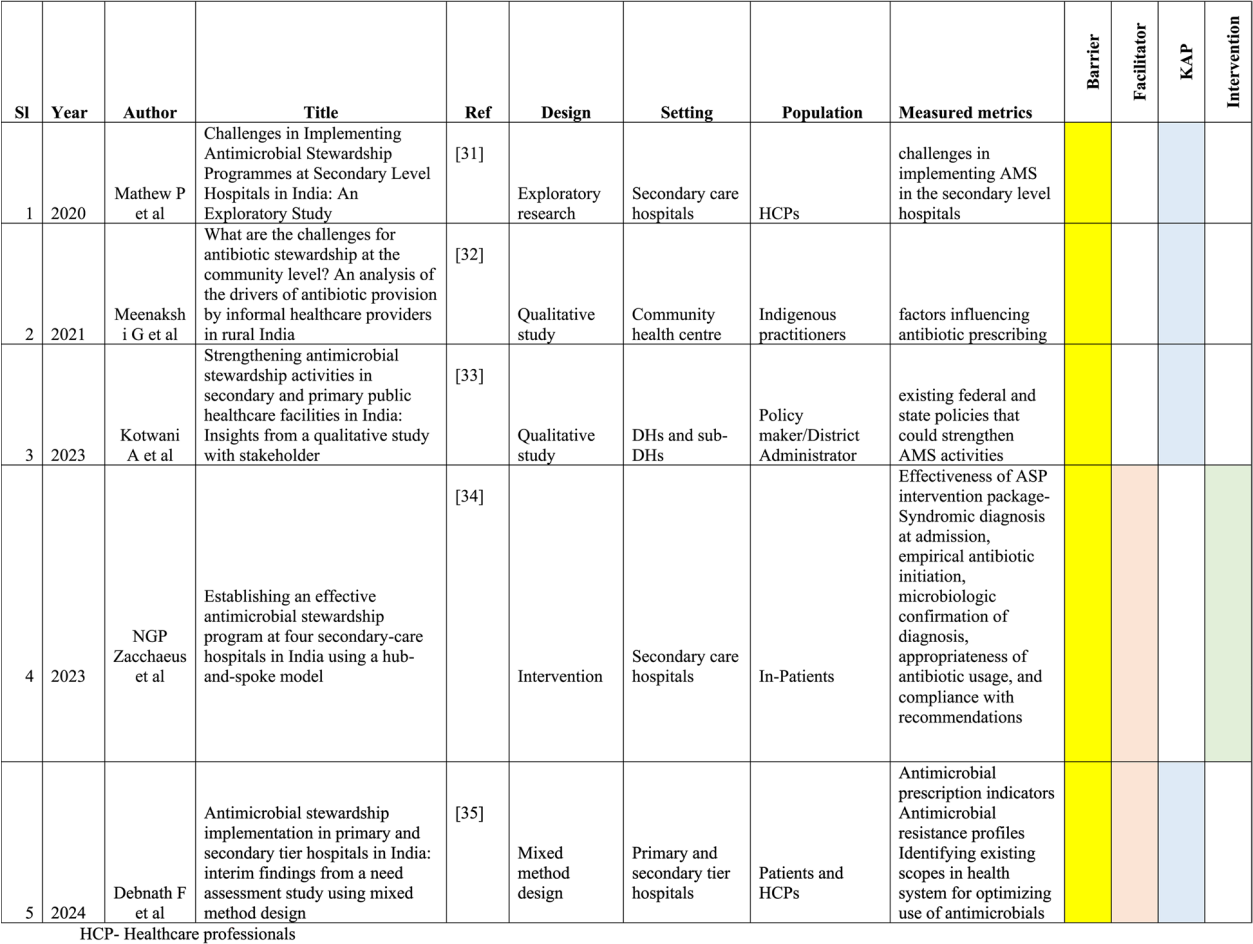
Characteristics of study considered for scoping review of antimicrobial stewardship in the Indian secondary and primary care clinical settings since 2015

**Table 2:** Significant barriers identified for scoping review of antimicrobial stewardship in the Indian secondary and primary care clinical settings since 2015

Sl	Year	Author	Major barriers identified
1	2023	Anita Kotwani et al. [33]	• Lack of monitoring of antimicrobial use • Non-adherence to STGs • Shortage of human resources • Lack/limited availability of Diagnostic microbiology laboratory services • Lack of DTC or functional DTC • Ineffective IPC practices • Lack of understanding the gravity of AMR by doctors and other HCPs • Lack of effective hospital leadership to monitor prescription • compliance for appropriate antimicrobial use
2	2023	NGP Zacchaeus et al. [34]	• Lack of training for healthcare professionals on judicious antibiotic prescribing • Inadequate laboratory diagnostic facilities and interpretation • Absence of antibiogram • Lack of antibiotic champion • Non-availability of standard treatment guidelines for infections
3	2020	Mathew P et al. [31]	• Minimal supporting facilities • Poor enforcement of regulations • Utility value of antibiogram • Competition among doctors • Absence of a champion • Time constraints • Lack of interdepartmental co-ordination
4	2021	Meenakshi G et al. [32]	• Limited knowledge on sale and use of antibiotics • Patients’ physical and economic needs • Drug promotional activities
5	2024	Debnath F et al. [35]	• Lack of scheduled training on AMR and AMSP • Lack of resources • Absence of motivation • Frequent transfer of trained staff to another facility • Lack of awareness on AMR • Not following available guideline, hence irrational antibiotic prescription

*STG *Standard Treatment Guidelines, *DTC* Drug and Therapeutics Committee, *IPC* Infection Prevention and Control, *HCP *Healthcare Professionals, *AMR* Antimicrobial Resistance, *AMSP* Antimicrobial Stewardship Program

**Table 3:** Challenges, physicians’ attitude and practice on AMSP of included studies in the scoping review of antimicrobial stewardship programs in Indian context

KAP of physicians and other healthcare professionals
Lack of Awareness and Knowledge
• Many clinicians are not fully aware of AMSP guidelines
• Limited understanding of the importance and impact of antimicrobial resistance
• Inadequate training on the correct use of antibiotics
Resistance to Change
• Some clinicians are resistant to changing established prescribing habits
• Belief that patient satisfaction requires prescribing antibiotics, even when not necessary
• Perception that AMSP implementation is an administrative burden rather than a clinical priority
Perception of Administrative and Logistical Support
• Belief that there is insufficient administrative and logistical support for AMSP
• Feeling that there are inadequate resources to effectively implement AMSP
Variable Adherence to Guidelines
• Inconsistent application of ASP guidelines in clinical practice
• Over-reliance on empirical therapy without adequate diagnostic confirmation
• Over-the-counter sale and prescription of antibiotics without proper oversight
Implementation of Periodic Training and Feedback
• Some hospitals have implemented periodic training and feedback mechanisms
• Use of electronic health records to track antibiotic prescriptions in some settings facilitate AMSP

**Table 4:** Significant impact of AMS intervention observed in the secondary care hospitals implemented AMSP in the Indian setting

Year	Author	Reference	Appropriateness of Antibiotic prescription	Antibiotic consumption	Clinical outcome	Microbiological outcome
2023	NGP Zacchaeus et al.	[34]	Dose optimisation	Reduced DOT per 1,000 patient days	Decreased length of stay	Significant reduction in the Prevalence of MDRO (ESBL, CRO, MRSA, VRE)
			antibiotic de-escalation			
			timing and duration of antibiotic prophylaxis		Decreased hospitalization days	
					Reduced hospital-acquired infections	

*DOT* Days of Therapy, *MDRO *Multidrug Resistant Organism, *ESBL* Extended Spectrum Beta-Lactamases, *CRO *Carbapenem-Resistant Organisms, *MRSA* Methicillin-Resistant *Staphylococcus aureus*, *VRE *Vancomycin-Resistant Enterococcus

### Study quality assessment

We attempted to conduct a quality appraisal to provide an insight into the robustness of the evidence in this area of research. We used the Critical Appraisal Skills Programme (CASP) checklist for the assessment of qualitative studies and Risk of Bias in Non-randomised Studies - of Interventions **(**ROBINS-1) for interventional study as these tools are designated to evaluate the methodological rigor of the respective studies. Three independent reviewers appraised the quality of the included studies using the selected tools. Discrepancies were resolved through discussion, and another researcher was consulted when necessary.

## Results

### Description of studies

**Fig. 1:**
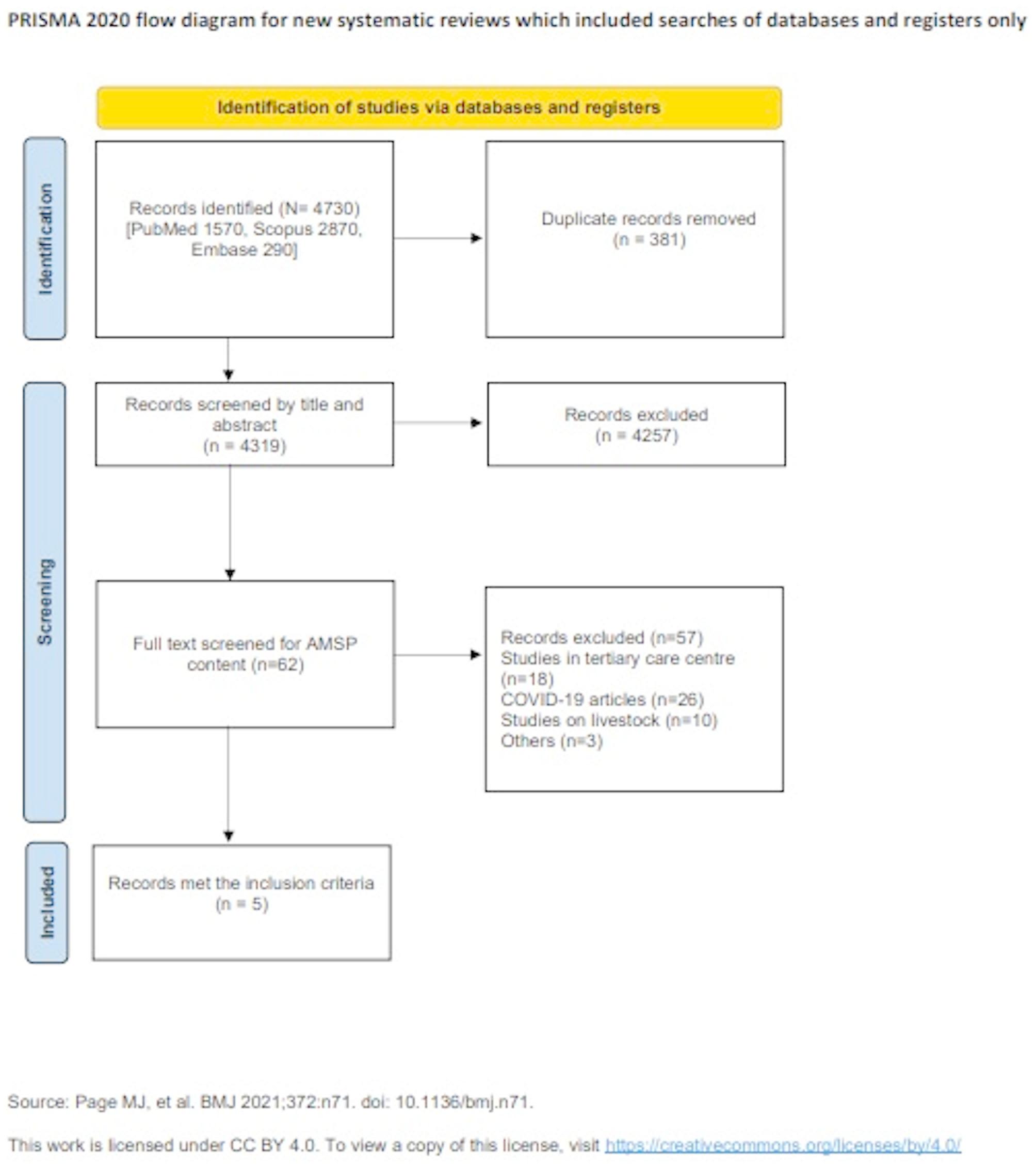
A PRISMA flowchart showing the studies chosen for the scoping review of antimicrobial stewardship initiatives in India, from 2015 to 2025

After duplicates were eliminated, 4319 of the 4730 papers that the literature search yielded were evaluated. Merely five articles were retained for the ultimate evaluation, after the removal of 4257 records from the first screening and 57 articles from the full-text screening. The PRISMA flow diagram shows the identification, screening, eligibility, and synthesis of findings processes (Fig. 1).

### Characteristics of the included studies

All the included studies reported barriers to the implementation of AMSP [31–35]. Similarly, four studies focused on prescriber perception and practice towards AMSP [31–33, 35]. Facilitators for AMSP implementation were addressed by two studies [34, 35], and another study examined the impact of AMSP implementation in established centers [34]. The studies were conducted across various regions of India, including Delhi, Madhya Pradesh, West Bengal, Assam, Tamil Nadu, Kerala, and Karnataka. This distribution reflects representation from the North, South, East, Central, and Northeastern regions of the country, thereby providing a diverse view of AMSP implementation across different healthcare settings. Regarding study design, the review included one pre-post study, two qualitative studies, one exploratory research and one mixed method study. All the studies were multicentric and were conducted between 2019 and 2023 (Table 1).

### Quality assessment of studies

Out of five studies, four studies were qualitative and subjected to quality assessment using CASP checklist which resulted in overall low risk of bias [31–33, 35]. The studies provided a clear aim and utilized an appropriate qualitative methodology (semi-structured interviews and in-depth interviews) to explore healthcare providers’ experiences with establishment of AMSP in the Indian secondary care settings. Data collection and analysis were rigorous, with clear thematic analysis and participant experiences. However, there is limited discussion on reflexivity, which may affect how the researcher’s perspective influenced the findings. The quality of one interventional study reported in the review was assessed using ROBINS-1 tool [34] since the intervention was applied across multiple hospitals with comparison of outcomes before and after the intervention. Overall, the study demonstrated a moderate risk of bias. Although the study demonstrated considerable reduction in the antimicrobial consumption, there is a high chance of potential bias associated with confounding and missing data, especially due to the variability across the study locations. This scoping review primarily attempted to map the breadth of research rather than critically examine the evidence, which may restrict the depth of the conclusions obtained even if a formal quality evaluation was carried out. Overall, the study findings are valuable for informing future antimicrobial stewardship interventions in similar settings. The quality assessment performed for the individual studies are given on *Supplementary file 2*.

### Barriers to implementing AMSP

The Table 2 summarizes the notable barriers to the implementation of AMSP in primary and secondary care settings in India. Among the five studies that documented barriers, all studies reported limited knowledge and lack of training among healthcare professionals regarding AMR and the concept of AMSP, leading to irrational antibiotic prescriptions [31–35]. Three *studies identified the absence of a dedicated antibiotic* champion or leadership commitment and the lack of supporting facilities as significant challenges in implementing AMSP [31–33]. Other documented barriers include the absence of standard treatment guidelines [31], a monitoring system for antimicrobial use [32], and a facility-specific antibiogram [32, 33]. The ‘miscellaneous’ category highlighted issues such as drug promotional activities, time constraints, and the physical and economic needs of patients, which compel clinicians to prescribe antibiotics without rational considerations [33, 34]. Additionally, lack of interdepartmental coordination, competition among doctors, and ineffective infection control practices were identified as barriers to the successful implementation of antibiotic stewardship in clinical settings [33].

### Facilitators to implement AMSP

Of the five studies identified, two studies discussed the enablers of AMSP. This study cited proper training and orientation for clinicians as one of the most prominent facilitators [34, 35]. Additionally, the establishment of a dedicated AMS team, timely access to laboratory facilities, and the prompt development of facility-specific antibiograms were recognized as potent enablers for effective AMSP implementation. An antibiogram, which is a summary document of antibacterial susceptibility patterns of local bacterial isolates, aids prescribers in selecting appropriate antimicrobials, determining local susceptibility rates, and tracking trends in antibiotic resistance over time. It also allows for comparisons between hospitals regarding antibiotic resistance trends [32]. In secondary care hospitals, coordination with tertiary care hospitals can facilitate successful AMS implementation [34]. Other facilitating factors include a prospective audit and feedback mechanism, the development of context-specific antimicrobial treatment guidelines, strict adherence to these guidelines, and periodic follow-ups [34, 35].

### Prescriber perception on AMSP implementation

Four out of five study designs were qualitative in nature and discussed the challenges for the implementation of AMSP in the primary and secondary care clinical settings [Table 3]. The prescriber perception and attitude on AMSP implementation was categorized into knowledge, attitude and practices (KAP). All four studies exclusively commented on the prescriber practice aspects related to AMS implementation in the secondary care settings [31–33, 35]. The pharmaceutical drug promotional activities are one of the prominent factors influencing the clinician to prescribe antibiotics, which is often called as continuing medical education (CME) program [31, 32]. Pharmaceutical companies sponsoring medical conferences and the regular visits of sales representatives, who provide drug samples and information increase the likelihood of subtly or overtly promoting such products, also antibiotics. In addition, the physical and economical needs of the patient also compel the physician to prescribe antibiotics, which may be inappropriate at times [33]. Two studies reported the sub-optimal knowledge of physicians leading to the irrational prescription of antibiotics [32, 33]. They often lack understanding about the sale and use of antibiotics and are unaware about the concept of AMR and AMSP. In terms of attitude, physicians and pharmacists, namely, showed positive response as they indicate it is beneficial for both clinician and the patient [31, 34]. However, conformism and lack of trust between the healthcare professionals challenge the implementation of AMSP in the secondary care settings [31]. In addition, physicians revealed a concern that their prescribing autonomy will be questioned by the implementation of AMSP [31, 32]. However, proper training provided to the healthcare professionals, formation of a dedicated AMS team, developing facility-specific treatment guidelines, adopting context-specific antibiogram, proper documentation and feedback might attain positive impact on AMSP implementation [31–35].

### Impact of AMSP implementation

The establishment of AMSP in secondary care hospitals was undertaken in a single study using a hub and spoke model, where a tertiary care hospital from South India acted as a facilitator for the implementation [34]. The impact of AMS interventions was reported in terms of the appropriateness of antibiotic prescriptions, antibiotic consumption, and clinical and microbiological outcomes. Metrics for measuring appropriateness included dose optimization, timely escalation or de-escalation of antibiotics, timing of initiation, and duration of treatment. The study reported a considerable reduction in antibiotic consumption following the initiation of tailored AMS interventions in the respective hospitals, measured as DOT per 1000 patient days. The length of hospital stays, hospital readmission rate, and the rate of HAIs measured patient-centered outcomes. Microbiological outcomes were assessed by the overall prevalence of MDRO, which showed a significant reduction in the post-intervention phase, including decreased prevalence of strains such as Extended Spectrum Beta-Lactamases (ESBL), Carbapenem-Resistant Organisms (CRO), Methicillin-Resistant *Staphylococcus aureus* (MRSA), and Vancomycin-Resistant Enterococcus (VRE). Economic outcomes were not assessed in the study, despite being considered one of the metrics for evaluating the impact of AMS interventions.

## Discussion

The major goal of this scoping review was to assess the state of hospital-based AMS therapies in the Indian context, with an emphasis on primary and secondary care settings. It concentrated on four major areas: challenges, facilitators, prescriber attitudes and behaviors, and the outcomes of AMS interventions in established centers. Based on the review, the most common challenges to AMSP implementation were found to be a lack of diagnostic facilities and a shortage of staff. On the other hand, the presence of hospital AMSP guidelines and specialized interdisciplinary teams served as important facilitators. The review also revealed a lack of understanding and perceptions regarding AMR and the AMSP concept among clinicians. Personalized AMSP interventions, on the other hand, were effective in encouraging the prudent use of antibiotics, lowering antibiotic usage, and lowering secondary infections acquired from the hospital.

Insufficient human resources, inadequate laboratory infrastructure, unreliable institutional antibiograms, disregard for national guidelines, financial constraints, and inadequate AMSP orientation and training are just a few of the challenges encountered when implementing AMSP in settings with limited resources [31–35]. These results are consistent with other research emphasizing the inadequate laboratory facilities for microbiology and the unreliability of antibiograms [1]. Physicians’ mistrust of microbiological results is a serious problem that frequently results in their preference for broad-spectrum empirical therapy based only on anecdotal evidence [1, 14, 25, 36]. The absence of context-specific antibiotic recommendations and AMSP training were two other significant challenges. Prescribers are unaware of the regional trends of AMR in the absence of guidelines and a reliable antibiogram, which is a reason for the overprescription of broad-spectrum antibiotics. Insufficient AMSP training results in limited understanding and practice of AMS principles among prescribers.

Facilitators for AMS implementation included the existence of hospital AMSP guidelines, which provide prescribers with the necessary tools to prescribe narrow-spectrum antibiotics. Readily available antibiograms offer physicians reliable local antibiotic resistance patterns, aiding in rational antibiotic prescribing [34]. The review documented those prescribers generally had a substandard level of knowledge about AMS principles [31–33]. This inadequate understanding can lead to irrational antimicrobial prescriptions. Despite this, studies reported positive perceptions towards AMSP, with physicians acknowledging that AMS interventions could encourage more careful consideration of antibiotic choices, which is beneficial in reducing AMR, length of hospitalization, and healthcare costs. Many prescribers were receptive to frequent monitoring and feedback on rational use of antibiotics and recommended tailored AMSP training. However, some studies also indicated that physicians were concerned about potential loss of prescribing autonomy due to AMSP implementation [31]. This concern has been commonly addressed in previous literature and is consistent with our study findings. AMSP interventions were found to improve rational antibiotic prescribing through dose optimization, antibiotic de-escalation, and a reduction in antibiotic prescriptions. Despite substantial heterogeneity in the metrics used to quantify antibiotic use and consumption, the most common metrics included DOT per 1000 patient-days. Clinical outcomes following AMSP implementation included reductions in hospital length of stay, in-hospital mortality, hospital readmission rates, and secondary infection rates. Positive clinical results of stewardship activities have also been confirmed by previous systematic reviews [37]. Positive microbiological outcomes were also observed, such as a reduction in the prevalence of MDROs and bacterial resistance [34]. These results show that AMSPs have a positive influence on microbiological outcomes, including a decline in the prevalence of bacterial and multi-drug resistance, and successfully decrease the needless use of antibiotics among hospital inpatients. These results also imply that ASPs accomplish their main goal of lowering unnecessary antibiotic use and related expenses without sacrificing clinical results.

None of the studies reported on economic outcomes assessed after the implementation of AMSP. According to previous literature, it has been reported on the economic impacts of AMSPs, a positive effect on cost reduction highlighting the financial benefits of ASP implementation in clinical settings but not present in our findings [34, 38].

The intervention study included lacked control groups and were non-randomized, making it challenging to control for confounding effects [34]. Other studies were qualitative in nature, limiting the generalizability of the findings [31–33, 35]. Multi-center trials, especially those conducted in rural areas, should be the focus of future efforts. The intervention trials did not take antibiotic cost reductions into account and had brief follow-up periods, which made it challenging to evaluate long-term effects on AMR trends, hospital readmissions, and death. Evidence of long-term economic benefits would persuade decision-makers and interested parties to fund AMSP initiatives. Infection prevention and control (IPC) programs should be integrated into AMSP to enhance patient safety and healthcare quality [39]. The WHO recommends the formation of multidisciplinary AMS teams comprising clinical pharmacists, microbiologists, infectious disease specialists, and nurses with IPC knowledge [40]. There is only one study that recorded the engagement of these teams and tried to create AMSP in secondary care hospitals in India [34]. To guarantee the sustainability of stewardship initiatives, future AMSP initiatives ought to focus on assembling diverse, interdisciplinary teams.

According to our findings, one of the immediate goals of AMS programs in India is to improve antibiotic prescribing practices, as antibiotics are frequently prescribed inappropriately in many healthcare settings due to a lack of knowledge or the pressure to provide quick fixes for infections [1, 31, 35]. This misuse and overuse of antibiotics significantly contribute to the rise of AMR, making it crucial to enhance the accuracy and appropriateness of antibiotic prescriptions to curb the spread of resistant infections. Achieving these goals in resource-constrained settings requires innovative and context-specific strategies that make the most of available resources. One effective strategy to enhance antibiotic prescribing practices is the implementation of Standard Treatment Guidelines (STGs). These guidelines, tailored to local contexts and prevalent infections, provide clear instructions for healthcare providers on when and how to prescribe antibiotics. Regular updates to these guidelines, based on local resistance patterns, ensure that they remain relevant and effective [41, 42]. Additionally, audit and feedback mechanisms can be employed to monitor and improve prescribing practices. By conducting periodic audits of antibiotic prescriptions and providing feedback to prescribers, healthcare facilities can encourage adherence to STGs and reduce inappropriate antibiotic use. Another essential goal is to strengthen IPC measures within healthcare facilities, as HAIs are common in many Indian hospitals and often lead to increased antibiotic use, further exacerbating AMR. By effectively implementing IPC measures and reducing HAIs, the demand for antibiotics can be significantly reduced. To strengthen IPC measures, healthcare facilities can focus on basic, cost-effective interventions that have a significant impact. These include promoting hand hygiene, ensuring the sterilization of medical equipment, and maintaining a clean environment within healthcare facilities [16, 43]. Existing nursing staff and infection control officers can be utilized to train and monitor adherence to these practices. Furthermore, developing and implementing surveillance programs to track infection rates and antibiotic use can help healthcare providers identify areas for improvement and target interventions more effectively. Even simple, paper-based or digital tools can be employed to collect and analyse data on HAIs, making surveillance feasible in resource-limited settings.

Besides, it is high time to raise awareness and educate healthcare providers, patients, and the broader community about AMR, as a lack of understanding of AMR and AMS practices hinders the successful implementation of stewardship programs. Educating all stakeholders on the importance of responsible antibiotic use and the danger of AMR is vital for the success of AMS initiatives. Increasing awareness and education on AMR requires ongoing efforts to train healthcare providers and educate the community. Regular training sessions for healthcare workers, integrated into routine clinical meetings and CME sessions, can help keep them informed about the latest developments in AMR and AMS practices [44, 45]. Several studies emphasize the importance of capacity building and continuous education for healthcare professionals as a critical component of effective AMSP implementation. Majority of the research done sustainable implementation of AMSP in the healthcare settings emphasized the importance of sustained educational interventions as a long-term strategy to address inappropriate antibiotic use [46]. However, detailed strategies on how to implement such interventions are often lacking. Educational interventions ranging from formal training sessions to ongoing professional development have been shown to positively influence prescribing behavior and increase adherence to stewardship principles. To ensure sustainable AMS practices, education should begin at the undergraduate level and continue through to professional and specialist training. This is consistent with the findings of an interventional study from our review that highlighted the role of structured, ongoing education as a critical long-term component of successful and sustainable implementation of AMSP [34]. Similarly, Chetty S et al. (2019) highlight the need for structured interprofessional AMS education to improve stewardship outcomes in the clinical settings [13]. In addition, a study by Mubarak N et al. (2021) revealed notable gaps in preparedness and confidence among medical and pharmacy students in managing antibiotic use and understanding resistance [47]. These findings underscore the need to embed AMS education early in healthcare training, and to reinforce it at every level of professional development particularly in regions like India, where general practice settings are often the first point of care. Additionally, community outreach programs can be leveraged to disseminate information on the prudent use of antibiotics and the dangers of AMR. Collaborating with local non-governmental organisations (NGOs) and community health workers can extend the reach of these educational initiatives, ensuring that even rural and underserved populations receive vital information [48, 49].

Expansion of AMSP in India’s primary and secondary care settings hinges on the active involvement of general physicians, who are often the first point of contact for patients. Several studies suggest that without their engagement, stewardship programs may struggle to create sustainable behavioral change in prescribing practices. Their involvement is not only crucial for identifying context-specific prescribing challenges but also for implementing sustainable stewardship practices [35]. However, most AMS initiatives in India have traditionally focused on tertiary care and hospital-based models, often overlooking the realities of general practice. To bridge this gap, there is a pressing need to develop collaborative frameworks, both theoretical and practical, that include general practitioners as key stakeholders. Without their engagement, meaningful and long-term implementation of AMS strategies may remain unattainable. The study by Mubarak N et al. (2021) underscores the importance of such collaboration, offering practical insights into how integration at the primary care level can influence AMS outcomes and guide future policy and practice. Including general physicians in AMS planning, training, and feedback loops will be essential for ensuring broad-based effectiveness across the healthcare system [48].

In the realm of healthcare, methodological strategies for example, design science approach are crucial, particularly for the effective and sustainable implementation of AMS initiatives. These approaches provide a structured, evidence-based framework for developing, implementing, and refining interventions that are both effective and practical in real-world settings [50–54]. Such approaches emphasize the creation of innovative, context-specific solutions through a systematic approach. By bridging the gap between theory and practice, these strategies ensure that interventions are not only scientifically sound but also adaptable and sustainable, leading to more effective outcomes in healthcare.

In order to give a thorough picture of the current status of AMSP in the Indian context, our scoping review gathered information from all four domains: barriers, facilitators, prescriber practice and behaviours, and the impact of AMS interventions. This review did, however, have certain shortcomings. To avoid linguistic issues, we only included publications published in English, which would have led to the removal of studies written in other languages. Additionally, we identified only one intervention study where AMSP was implemented and assessed for outcomes. Although this was a multicentric study, generalizing its findings is challenging. Moreover, the scope of our review was limited to Indian studies published between 2015 and 2023, which might have led to the exclusion of relevant studies published earlier. We chose to include articles from 2015 onwards because the WHO launched the GAP to mitigate AMR that year. This approach provides more recent data, which is valuable for planning methodological strategies for the effective implementation of AMSP tailored to clinical settings. Lastly, we did not address potential biases in the selected studies, and we excluded commentaries, reviews, study reports, and expert opinions.

## Conclusion

India’s rapid growth in population and development poses unique challenges in addressing antimicrobial resistance (AMR), a pressing global public health concern. Our findings, focused on secondary and primary care hospitals in India, highlight the need for targeted interventions to establish antimicrobial stewardship programs (AMSP) in these settings. The review suggests that strengthening capacity-building programs, improving basic infrastructure, and enhancing workforce competencies are essential steps toward mitigating the AMR crisis. Tailoring AMSP to resource-constrained settings can support the rational use of antimicrobials and contribute to better health outcomes for vulnerable populations.

Future AMSP initiatives should focus on reporting microbiological, clinical, and economic outcomes to provide comprehensive evidence of their impact. Organizational support and the development of facility-specific treatment guidelines are pivotal to the successful implementation of AMSP. While knowledge and awareness of AMR and AMSP among healthcare professionals remain limited, their positive attitudes and willingness to engage in training and mentorship programs highlight opportunities for progress. Customizing AMS interventions to specific clinical settings, leveraging existing resources, and fostering collaborations with tertiary care centres can significantly enhance AMS activities in primary and secondary care hospitals.

By prioritizing the formation of multidisciplinary AMS teams, integrating infection prevention and control (IPC) efforts, and developing facility-specific AMS policies, India can take meaningful strides toward addressing AMR in these critical healthcare settings. These tailored strategies have the potential to improve antibiotic use, reduce unnecessary consumption, and achieve better patient-centered and microbiological outcomes, ultimately contributing to a more effective response to the AMR challenge.

### Study limitations

The study has certain limitations. First, this scoping review consists of only five studies that cannot be generalized for the findings. The shortage of studies in AMSP in the primary and secondary clinical care settings in the Indian context reflects a huge requirement of research in that area. In addition, the five studies varied in methodology and quality which may result in significant heterogeneity in the results. Second, the scoping review had language restrictions where articles published in English language was only considered for review that may introduce publication bias and limit the comprehensiveness of the review. Also, the geographical scope was restricted to India and was not interested in any other LMICs with similar healthcare challenges. Third, the studies published after 2015 were considered, following WHO Global Action Plan. Since this ensures the inclusion of recent data, there is a chance of exclusion of earlier studies with potential insights. Finally, scoping reviews have limitations to critically appraise the quality of evidence or in establishing causal relationships. Still, we attempted to perform a quality appraisal of the included studies, with the primary goal of mapping the existing evidence. However, the scope of our conclusions was limited by this aim, as we sought to understand the extent of AMS implementation programs in primary and secondary care clinical settings in India. This is crucial for preventing drug-resistant pathogens in tertiary care settings, as the majority of cases are referred from primary or secondary care. Future systematic review and meta-analysis are required that include a greater number of studies with robust quality appraisal to validate and provide more insights on the topic.

## Supplementary Information

Supplementary Material 1.

Supplementary Material 2.

Supplementary Material 3.

Supplementary Material 4.

Supplementary Material 5.

Supplementary Material 6.

## Data Availability

The datasets used and/or analysed during the current study are available from the corresponding author on reasonable request. No additional datasets were generated during the study.
