# Supplementary material for: Antimicrobial stewardship programs in primary and secondary care settings in India: current challenges, facilitators, perceptions, and impact – a scoping review
Source: BMC Infect Dis. 2025 Nov 11;25:1539. doi: 10.1186/s12879-025-11851-0 (PMC12606880; doi:10.1186/s12879-025-11851-0)
Supplement: Supplementary file 5 — Supplementary Material 5. [file 12879_2025_11851_MOESM5_ESM.docx]

**The detailed search strategy in different databases**

1. PubMed Search strategy

| **Search** | **Query** | **Items found** |
| --- | --- | --- |
|  | (((((((((((Drug Resistances, Microbial) OR (Antibiotic Resistance, Microbial)) OR (Antibiotic Resistance)) OR (Resistance, Antibiotic)) OR (Antimicrobial Drug Resistance)) OR (Antimicrobial Drug Resistances)) OR (Antimicrobial Resistance, Drug)) OR (Antimicrobial Resistances, Drug)) OR (Drug Antimicrobial Resistance)) OR (Drug Antimicrobial Resistances)) OR (Resistance, Drug Antimicrobial)) OR (Resistances, Drug Antimicrobial) | 2,83,058 |
|  | Search ((Stewardship, Antimicrobial) OR (Antibiotic Stewardship)) OR (Stewardship, Antibiotic) | 15,698 |
|  | Search ((((((((((((((((((((((Care Center, Secondary) OR (Care Centers, Secondary)) OR (Center, Secondary Care)) OR (Centers, Secondary Care)) OR (Secondary Care Center)) OR (Secondary Care Facility)) OR (Care Facilities, Secondary)) OR (Care Facility, Secondary)) OR (Facilities, Secondary Care)) OR (Facility, Secondary Care)) OR (Secondary Care Facilities)) OR (Secondary Referral Center)) OR (Center, Secondary Referral)) OR (Centers, Secondary Referral)) OR (Referral Center, Secondary)) OR (Referral Centers, Secondary)) OR (Secondary Referral Centers)) OR (Secondary Referral Hospital)) OR (Hospital, Secondary Referral)) OR (Hospitals, Secondary Referral)) OR (Referral Hospital, Secondary)) OR (Referral Hospitals, Secondary)) OR (Secondary Referral Hospitals) | 80,891 |
|  | Search (((((Care, Primary Health) OR (Health Care, Primary)) OR (Primary Care)) OR (Care, Primary)) OR (Primary Healthcare)) OR (Healthcare, Primary) | 7,03,950 |
|  | Search ((Rural Hospitals) OR (Hospital, Rural)) OR (Rural Hospital) | 62,728 |
|  | Search ((Community Hospital) OR (Community Hospitals)) OR (Hospital, Community) | 2,89,914 |
|  | Search Practice, General | 2,85,153 |
|  | ((((#3) OR (#4)) OR (#5)) OR (#6)) OR (#7) | 12,35,582 |
|  | ((#1) AND (#2)) AND (#8) | 1,704 |
|  | Limit to “2015-2025” | 1,570 |

1. SCOPUS Search Strategy

| **Search** | **Query** | **Items found** |
| --- | --- | --- |
| #1 | ( ALL ( "Drug Resistances, Microbial" ) OR ALL ( "Antibiotic Resistance, Microbial" ) OR ALL ( "Antibiotic Resistance" ) OR ALL ( "Resistance, Antibiotic" ) OR ALL ( "Antimicrobial Drug Resistance" ) OR ALL ( "Antimicrobial Drug Resistances" ) OR ALL ( "Antimicrobial Resistance, Drug" ) OR ALL ( "Antimicrobial Resistances, Drug" ) OR ALL ( "Drug Antimicrobial Resistance" ) OR ALL ( "Drug Antimicrobial Resistances" ) OR ALL ( "Resistance, Drug Antimicrobial" ) OR ALL ( "Resistances, Drug Antimicrobial" ) ) | 491306 |
| #2 | ( ALL ( "stewardship, antimicrobial" ) OR ALL ( "antibiotic stewardship" ) OR ALL ( "stewardship, antibiotic" ) ) | 17766 |
| #3 | ( ALL ( "care center, secondary" ) OR ALL ( "care centers, secondary" ) OR ALL ( "center, secondary care" ) OR ALL ( "centers, secondary care" ) OR ALL ( "secondary care center" ) AND ALL ( "secondary care facility" ) OR ALL ( "care facilities, secondary" ) OR ALL ( "care facility, secondary" ) OR ALL ( "facilities, secondary care" ) OR ALL ( "facility, secondary care" ) OR ALL ( "secondary care facilities" ) OR ALL ( "secondary referral center" ) OR ALL ( "center, secondary referral" ) OR ALL ( "centers, secondary referral" ) OR ALL ( "referral center, secondary" ) OR ALL ( "referral centers, secondary" ) OR ALL ( "secondary referral centers" ) OR ALL ( "secondary referral hospital" ) OR ALL ( "hospital, secondary referral" ) OR ALL ( "hospitals, secondary referral" ) OR ALL ( "referral hospital, secondary" ) OR ALL ( "referral hospitals, secondary" ) OR ALL ( "secondary referral hospitals" ) ) | 3215 |
| #4 | (ALL (“care, primary health”) OR ALL ( "care, primary health" ) OR ALL ( "primary care”) OR ALL (“care, primary”) OR ALL (“primary healthcare”) OR ALL (“healthcare, primary”)) | 1089442 |
| #5 | (ALL (“rural hospitals”) OR ALL (“hospital, rural”) OR ALL (“rural hospital”)) | 24532 |
| #6 | (ALL (“community hospital”) OR ALL (“community hospitals”) OR ALL (“hospital, community”)) | 101862 |
| #7 | ALL (“practice, general”) | 2852 |
| #8 | Search ((((#3) OR (#4)) OR (#5)) OR (#6)) OR (#7) | 1203362 |
| #9 | Search (#1) AND (#2) AND (#8) | 3061 |
| #10 | Limit to “2015-2025” | 2870 |

1. Embase Search Strategy

| **Search** | **Query** | **Items found** |
| --- | --- | --- |
| #1 | 'antibiotic resistance'/exp OR 'antibiotic resistance' | 269567 |
| #2 | ‘antimicrobial stewardship’/exp OR ‘antimicrobial stewardship’ | 19433 |
| #3 | ‘secondary care center’/exp OR ‘secondary care center’ | 2914 |
| #4 | ‘public health service’/exp OR ‘public health service’ | 94142 |
| #5 | ‘rural hospital’/exp OR ‘rural hospital’ | 5168 |
| #6 | ‘community care’/exp OR ‘community care’ | 157343 |
| #7 | ‘general practice’/exp OR ‘general practice’ | 164343 |
| #8 | Search ((((#3) OR (#4)) OR (#5)) OR (#6)) OR (#7) | 412301 |
| #9 | Search (#1) AND (#2) AND (#8) | 290 |
