# Supplementary material for: Antimicrobial stewardship programs in primary and secondary care settings in India: current challenges, facilitators, perceptions, and impact – a scoping review
Source: BMC Infect Dis. 2025 Nov 11;25:1539. doi: 10.1186/s12879-025-11851-0 (PMC12606880; doi:10.1186/s12879-025-11851-0)
Supplement: Supplementary file 6 — Supplementary Material 6. [file 12879_2025_11851_MOESM6_ESM.pdf]

CASP Checklist:
For Qualitative Research

**Reviewer Name:**	Anonymous
**Paper Title:**	Antimicrobial stewardship implementation in primary and secondary tier hospitals in India: interim findings from a need assessment study using mixed method design
**Author:**	Falguni Debnath,<br>Rajyasree Ghosh De,<br>Debjit Chakraborty,<br>Agniva Majumdar,<br>Sandip Mukhopadhyay,<br>Munmun Das Sarkar,<br>Taru Singh,<br>Sanjit Kumar Patra,<br>Surangana Saha,<br>Julius Rehman,<br>Dhiraj Roy,<br>Atreyi Chakrabarti,<br>Sulagna Basu,<br>Asish Kumar Mukhopadhyay,<br>Amitabha Mondal,<br>Shyamal Soren,<br>Kalpana Datta,<br>Shantasil Pain,

	Supreeti Biswas Mondal,<br>Palash Mondal,<br>Kamini Walia,<br>Dipankar Maji,<br>Alok Kumar Deb &<br>Shanta Dutta
**Web Link:**	https://doi.org/10.1038/s41598-024-78111-0
**Appraisal Date:**	07-04-2025

During critical appraisal, never make assumptions about what the researchers have done. If it is not possible to tell, use the "Can't tell" response box. If you can't tell, at best it means the researchers have not been explicit or transparent, but at worst it could mean the researchers have not undertaken a particular task or process. Once you've finished the critical appraisal, if there are a large number of "Can't tell" responses, consider whether the findings of the study are trustworthy and interpret the results with caution.

Section A Are the results valid?	
1. Was there a clear statement of the aims of the research?	☒Yes ☐ No ☐ Can't Tell<br><br>(1) To calculate antimicrobial prescription rate (APR), multiple antimicrobial prescription rate (MPR) for common infections in primary and secondary tier hospitals.<br>(2) To calculate the proportion of matching of empirical treatment with antibiogram of isolated organisms from common illnesses in primary and secondary tier hospitals.<br>(3) To understand the existing scope of health system for optimizing use of antimicrobials for common infections.
CONSIDER:<br>• *what was the goal of the research?*<br>• *why was it thought important?*<br>• *its relevance*	
2. Is a qualitative methodology appropriate?	☒Yes ☐ No ☐ Can't Tell

	To meet the last objective, we collected qualitative data from medical officers, medical officers in administrative position, infection control nurses
CONSIDER:<br>• *If the research seeks to interpret or illuminate the actions and/or subjective experiences of research participants*<br>• *Is qualitative research the right methodology for addressing the research goal?*	
3. Was the research design appropriate to address the aims of the research?	☒Yes ☐ No ☐ Can't Tell<br><br>The study used an exploratory design with interviews or focus groups, this would be appropriate to uncover the in-depth challenges in implementing ASPs. However, if the design is purely descriptive without engagement with key stakeholders, it might limit the depth of understanding
CONSIDER:<br>• *if the researcher has justified the research design (e.g., have they discussed how they decided which method to use)*	
4. Was the recruitment strategy appropriate to the aims of the research?	☒Yes ☐ No ☐ Can't Tell<br><br>They examined 600 prescriptions from outpatient departments as per WHO guideline. Data on Prescriptions were collected through consecutive sampling on randomly selected days from different clinical disciplines such as Medicine, Paediatrics, General Outpatient Dept (OPD).<br>They collected qualitative data through eight in-depth interviews from required members such as medical officers, medical officers in administrative position, infection control nurses.
CONSIDER:<br>• *If the researcher has explained how the participants were selected*<br>• *If they explained why the participants they selected were the most appropriate to provide access to the type of knowledge sought by the study*<br>• *If there are any discussions around recruitment (e.g. why some people chose not to take part)*	
5. Was the data collected in a way that addressed the research issue?	☒Yes ☐ No ☐ Can't Tell<br><br>The study conducted interviews with medical officers, administrative officers, and infection control nurses. These interviews aimed to identify existing strengths and potential facilitators for implementing Antimicrobial Stewardship Programs (AMSP) in lower-tier hospitals.
CONSIDER:<br><br>• *If the setting for the data collection was justified*<br>• *If it is clear how data were collected (e.g. focus group, semi-structured interview etc.)*<br>• *If the researcher has justified the methods chosen*	

• *If the researcher has made the methods explicit (e.g. for interview method, is there an indication of how interviews are conducted, or did they use a topic guide)*<br>• *If methods were modified during the study. If so, has the researcher explained how and why*<br>• *If the form of data is clear (e.g. tape recordings, video material, notes etc.)*<br>• *If the researcher has discussed saturation of data*	
6. Has the relationship between researcher and participants been adequately considered?	☐Yes ☐ No ☒ Can't Tell<br><br>The study involved interactions between researchers and participants, primarily through in-depth interviews with medical officers, administrative officers, and infection control nurses. However, the available information does not provide explicit details on how the relationship between researchers and participants was managed or considered during the study. Specifics regarding measures to address potential biases, power dynamics, or ethical considerations in these interactions are not detailed in the provided sources.
CONSIDER:<br>• *If the researcher critically examined their own role, potential bias and influence during (a) formulation of the research questions (b) data collection, including sample recruitment and choice of location*<br>• *How the researcher responded to events during the study and whether they considered the implications of any changes in the research design*	
Section B: What are the results?	
7. Have ethical issues been taken into consideration?	☒Yes ☐ No ☐ Can't Tell<br><br>The study acknowledges that the authors declare no competing interests, specific information regarding ethical approvals, informed consent processes, and measures to ensure participant confidentiality is provided in the accessible summaries. Given the study's involvement of human participants and data collection from medical records, it is standard practice to obtain approval from an institutional ethics committee and secure informed consent from participants.
CONSIDER:<br>• *If there are sufficient details of how the research was explained to participants for the reader to assess whether ethical standards were maintained*<br>• *If the researcher has discussed issues raised by the study (e.g. issues around informed consent or confidentiality or how they have handled the effects of the study on the participants during and after the study)*<br>• *If approval has been sought from the ethics committee*	
8. Was the data analysis sufficiently rigorous?	☐Yes ☐ No ☒ Can't Tell<br><br>The integration of both quantitative and qualitative data provided a comprehensive understanding of antimicrobial use and the contextual factors influencing AMSP

	implementation in these settings. However, the available information does not specify whether statistical measures, such as confidence intervals or significance testing, were employed to validate the quantitative findings. The qualitative analysis appears to have been conducted systematically, but detailed descriptions of coding processes or triangulation methods are not provided in the accessible sources.
CONSIDER:<br>• *If there is an in-depth description of the analysis process*<br>• *If thematic analysis is used. If so, is it clear how the categories/themes were derived from the data*<br>• *Whether the researcher explains how the data presented were selected from the original sample to demonstrate the analysis process*<br>• *If sufficient data are presented to support the findings*<br>• *To what extent contradictory data are taken into account*<br>• *Whether the researcher critically examined their own role, potential bias and influence during analysis and selection of data for presentation*	
9. Is there a clear statement of findings?	☒Yes ☐ No ☐ Can't Tell<br><br>The study concludes that routine infection control activities in lower-tier hospitals are currently delinked from antimicrobial resistance (AMR) containment measures. Therefore, it recommends the establishment of customized AMSPs in these hospitals, which cater to two-thirds of India's population.<br>These findings are presented clearly, with supporting data and analysis, providing a comprehensive understanding of the current state of antimicrobial stewardship in primary and secondary tier hospitals in India.
CONSIDER:<br>• *If the findings are explicit*<br>• *If there is adequate discussion of the evidence both for and against the researcher's arguments*<br>• *If the researcher has discussed the credibility of their findings (e.g. triangulation, respondent validation, more than one analyst)*<br>• *If the findings are discussed in relation to the original research question*	
Section C: Will the results help locally?	
10. How valuable is the research?	☒Yes ☐ No ☐ Can't Tell<br><br>This research is highly valuable as it sheds light on an often-overlooked segment of the healthcare system, provides actionable insights, and offers a pathway for extending antimicrobial stewardship efforts to a broader population base.
CONSIDER:<br>• *If the researcher discusses the contribution the study makes to existing knowledge or understanding (e.g., do they consider the findings in relation to current practice or policy, or relevant research-based literature*<br>• *If they identify new areas where research is necessary*	

- *If the researchers have discussed whether or how the findings can be transferred to other populations or considered other ways the research may be used*

**APPRAISAL SUMMARY**: *List key points from your critical appraisal that need to be considered when assessing the validity of the results and their usefulness in decision-making.*		
**Positive/Methodologically sound**	**Negative/Relatively poor methodology**	**Unknowns**
Clear Research Aim<br>Appropriate Methodology<br>Relevant Participant Selection<br>Ethical Approval<br>Findings Linked to Data<br>Practical Relevance	Limited Reflexivity<br>Underreported Analysis Process<br>Transferability Constraints	Depth of Data Saturation<br>Role of Participants in Validating Results<br>Long-Term Impact

Referencing recommendation:

CASP recommends using the Harvard style referencing, which is an author/date method. Sources are cited within the body of your assignment by giving the name of the author(s) followed by the date of publication. All other details about the publication are given in the list of references or bibliography at the end.

Example:

Critical Appraisal Skills Programme (2024). CASP (insert name of checklist i.e. systematic reviews with meta-analysis of randomised controlled trials (RCTs) Checklist.) [online] Available at: insert URL. Accessed: insert date accessed.

Creative Commons

©CASP this work is licensed under the Creative Commons Attribution – Non-Commercial- Share A like. To view a copy of this licence, visit https://creativecommons.org/licenses/by-nc-sa/4.0/

Need further training on evidence-based decision making? Our online training courses are helpful for healthcare educational researchers and any other learners who:

- Need to critically appraise and stay abreast of the healthcare research literature as part of their clinical duties.
- Are considering carrying out research & developing their own research projects.
- Make decisions in their role, whether that be policy making or patient facing.

Benefits of CASP Training:

⇒ Affordable – courses start from as little as £6
⇒ Professional training – leading experts in critical appraisal training
⇒ Self-directed study – complete each course in your own time
⇒ 12 months access – revisit areas you aren't sure of and revise
⇒ CPD certification - after each completed module

Scan the QR code below or visit https://casp-appraisal-online-uk.net/critical-training-courses/ for more information and to start learning more.
